# Prolonged allograft survival in liver transplantation –time to test the limits…pair with 24–00296

**DOI:** 10.1016/j.sopen.2025.04.001

**Published:** 2025-04-22

**Authors:** Alana Hofmann, Shimul A. Shah

**Affiliations:** a Department of Transplant Surgery, University of Cincinnati, Cincinnati, OH, United States of America; b Chief, Transplant Surgery, Mass General Brigham, Boston, MA, United States of America

Thank you to the team with Christine Hwang et al. of UT Southwestern Medical Center for using the UNOS STAR file to calculate the overall incidence of older liver allografts and allograft survival. Key understanding from this article is the matching of donor with the recipient and how this is an example of OPTN modernization that needs to occur in the future. In this manuscript, the reoccurring theme is older allografts need a lower MELD and healthier recipient at time of transplant to allow for the nature of the older graft slow initial function. Previous papers have examined the overall recipient population that does not do well, including higher MELDs, previous liver transplants, and portal vein thrombus [1]. The definition of “healthier” recipient has been described as first time, non-Status 1, age > 45, BMI <35, indication for transplant other than hepatitis C, and cold ischemic time < 8 hours [2]. As the transplant community tries to utilize these octogenarian/nonagenarian allografts, many centers have realized that older allografts can be placed into many different types of recipients making up 43 % of the waitlist [3]. The area to focus on with this manuscript is the overall long-term data analysis that shows the donor age plus donor-recipient survival of the older grafts are comparable to ideal or average liver allografts when considering the recipient's comorbidities. The future of this paper is the application of machine perfusion and increased cold ischemic times as more centers will likely be transitioning to older allografts given the donor demographic shifts occurring now. Will this affect recipient outcomes? Will there may management changes for older donors and how they are allocated? The findings in the paper set the stage to understand what our limits may be with things like machine perfusion, normothermic regional perfusion and organ allocation changes for hard to place organs.

## CRediT authorship contribution statement

**Alana Hofmann:** Writing – original draft. **Shimul A. Shah:** Writing – review & editing.

## Declaration of competing interest

No interests to declare for this manuscript.
